# Evaluation of immunomodulatory potential of probiotic conditioned medium on murine macrophages

**DOI:** 10.1038/s41598-024-56622-0

**Published:** 2024-03-26

**Authors:** Mohammad A. A. Al-Najjar, Shaymaa B. Abdulrazzaq, Lujain F. Alzaghari, Asma Ismail Mahmod, Amin Omar, Eliza Hasen, Tamara Athamneh, Wamidh H. Talib, Dinesh Kumar Chellappan, Muna Barakat

**Affiliations:** 1 Faculty of Pharmacy, Applied Science Private University, 11937 Amman, Jordan; 2 MEA Research Center, Middle East University, Amman, Jordan; 3 Institute of Nanotechnology, Jordan University of Science and Technology, Irbid, Jordan; 4 Department of Life Sciences, School of Pharmacy, International Medical University, 57000 Bukit Jalil, Kuala Lumpur, Malaysia; 5 Faculty of Allied Medical Sciences, Applied Science Private University, 11937 Amman, Jordan

**Keywords:** Probiotic, Macrophage, Immunity, Immunostimulatory, Phagocytosis, Proinflammatory, Immunology, Microbiology

## Abstract

Probiotics are a mixture of beneficial live bacteria and/or yeasts that naturally exist in our bodies. Recently, numerous studies have focused on the immunostimulatory effects of single-species or killed multi-species probiotic conditioned mediums on macrophages. This study investigates the immunostimulatory effect of commercially available active, multi-species probiotic conditioned medium (CM) on RAW264.7 murine macrophages. The probiotic CM was prepared by culturing the commercially available probiotic in a cell-culture medium overnight at 37 °C, followed by centrifugation and filter-sterilization to be tested on macrophages. The immunostimulatory effect of different dilution percentages (50%, 75%, 100%) of CM was examined using the MTT assay, proinflammatory cytokine (tumor necrosis factor TNF-alpha) production in macrophages, migration, and phagocytosis assays. For all the examined CM ratios, the percentages of cell viability were > 80%. Regarding the migration scratch, TNF-alpha and phagocytosis assays, CM demonstrated a concentration-dependent immunostimulatory effect. However, the undiluted CM (100%) showed a significant (*p*-value < 0.05) stimulatory effect compared to the positive and negative controls. The findings suggest that the secretions and products of probiotics, as measured in the CM, may be closely associated with their immune-boosting effects. Understanding this relationship between probiotic secretions and immune function is crucial for further exploring the potential benefits of probiotics in enhancing overall health and well-being.

## Introduction

Macrophages are recognized as significant participants in the response to acute infection, playing a crucial role in host immunity modulation and defending physiological integrity^1–3^. They perform vital functions in innate immunity, inflammation reactions, foreign antigen presentation, and dead cell scavenging^4^. Moreover, macrophages secrete immunoregulatory mediators, thereby facilitating an adaptive immune response to protect the host from foreign pathogens^5,6^. They protect the host from invasive pathogens by releasing inflammatory molecules such as nitric oxide (NO) and reactive oxygen species (ROS) and by secreting proinflammatory cytokines, such as interleukin-6 (IL-6) and tumor necrosis factor-α (TNF-α)^7,8^. Emerging evidence shows that these metabolites are essential for the immunostimulatory roles, and changes in their levels can affect the inflammatory status of macrophages. The cytokines secreted by macrophages can alter the properties of different cell types and provide necessary communication signals for motile cells and the immune system^9^. An uncontrolled shift toward macrophage activation is likely to result in autoimmune disorders and tissue damage. An imbalance in macrophage activation could reduce an organism's ability to combat infectious pathogens^2^. In turn, activated macrophages increase phagocytosis, secrete immunoregulatory cytokines, and enhance the production of cytotoxic molecules to destroy foreign pathogens^10^. Hence, macrophages are considered key target cells for immunomodulatory mechanisms.

Probiotics, which are part of the typical gut microbiota, support a balanced immune system^11^ and have a role in modulating human health and immune function^12^. The potential effects of probiotic bacteria on the immune system are increasingly attracting research interest for potential therapeutic and prophylactic applications in various disorders, ranging from diarrhea to allergies^13^. Certainly, many studies have reported their effectiveness in promoting overall health^14–16^. Daily intake of probiotic supplements has been proven to increase innate immune functions by significantly boosting IgA antibody levels and the number of phagocytic cells in the blood^17,18^. The ability of probiotics to regulate phagocytic cell activity may enhance their engulfment capacity and ability to kill harmful bacteria^19–21^. In addition, an extensive amount of evidence shows that *Lactobacillus* species have the ability to enhance the secretion of cytokines involved in innate immunity, such as IFN-γ, TNF-α, IL-18 and IL-12, in vitro in human peripheral blood mononuclear cells^22^. Probiotics have numerous health benefits, but little is known about their immunostimulatory activities and molecular mechanisms. Lactobacillus strains of probiotics have been clinically reported to reduce diarrhea caused by intestinal problems^23^. Furthermore, compelling evidence shows that secretory components of Lactobacillus may beneficially affect host immune activities^24–26^.

So far, to the best of our knowledge, no study has investigated the immunostimulatory effect of "active" multi-species probiotic conditioned medium on RAW264.7 murine macrophages. Almost all studies in this field have investigated single-species probiotics^27,28^, the effect of a killed multi-species probiotic conditioned medium^29^. As such, in this study, we evaluated the effect of cell-free Probiotic Conditioned Medium (CM) using Advanced Multi-Billion Dophilus commercially available probiotics, which are mainly Lactobacilli and Bifidobacteria. These bacteria have been reported to stimulate and regulate various aspects of both innate and acquired immune responses^30–33^ on RAW264.7 RAW264.7 macrophages, strategies that are critical for enhancing and protecting immune function.

## Results

### Effect of CM on RAW264.7 murine macrophage viability

**Figure 1:**
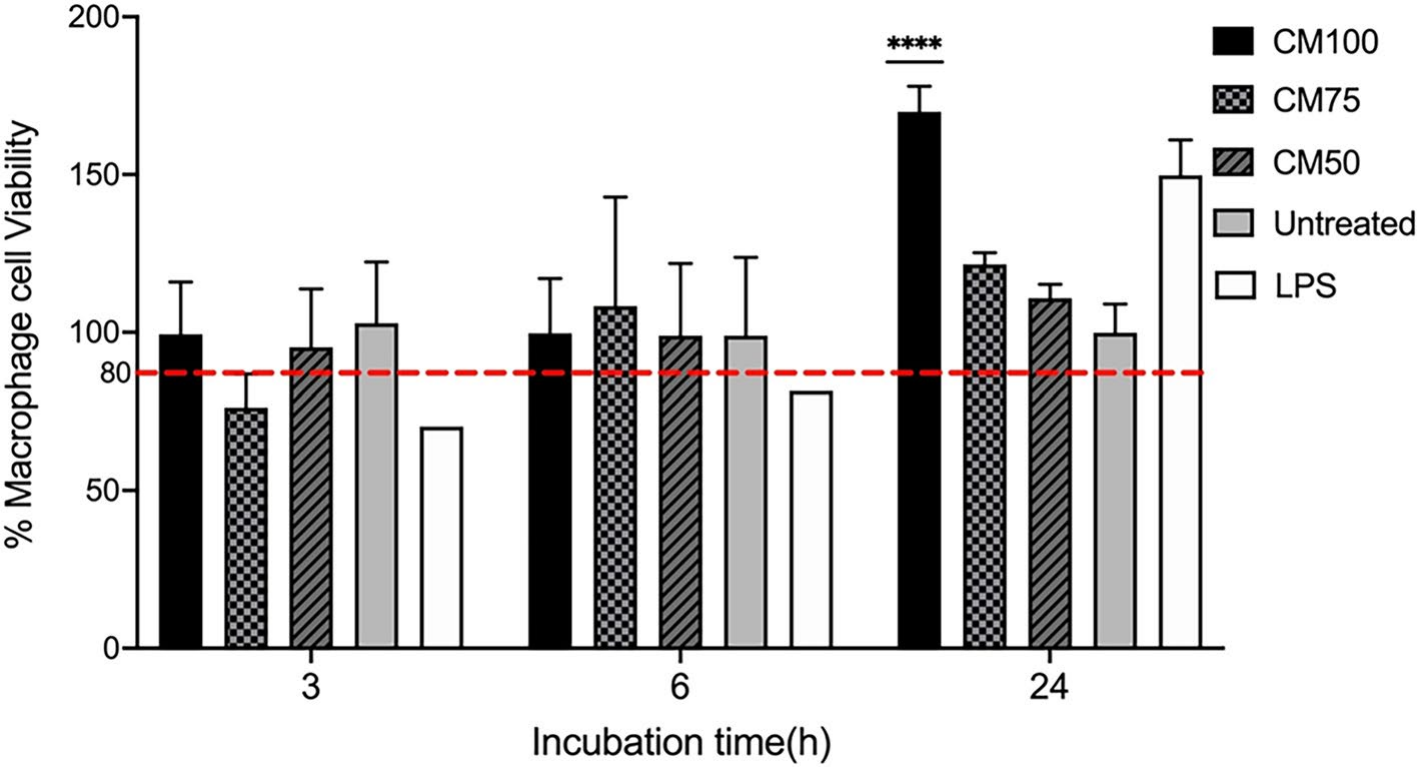
The percentage of viable RAW264.7 murine macrophages (in triplicates) over 3, 6, and 24h timeframes is shown with conditioned media CM100 for Probiotic Conditioned Media concentration 100%, CM75 for Probiotic Conditioned Media concentration 75%, and CM50 for Probiotic Conditioned Media concentration 50%. The effects of CM of different concentrations are compared to a control LPS (lipopolysaccharide at 1µg/mL). Statistics were performed using a one-way ANOVA test, comparing the groups with the control. Data are presented as means with standard deviations. Level of significance is denoted as follows: *p < 0.05, **p < 0.001, ***p < 0.001, and ****p < 0.0001.

**Figure 2:**
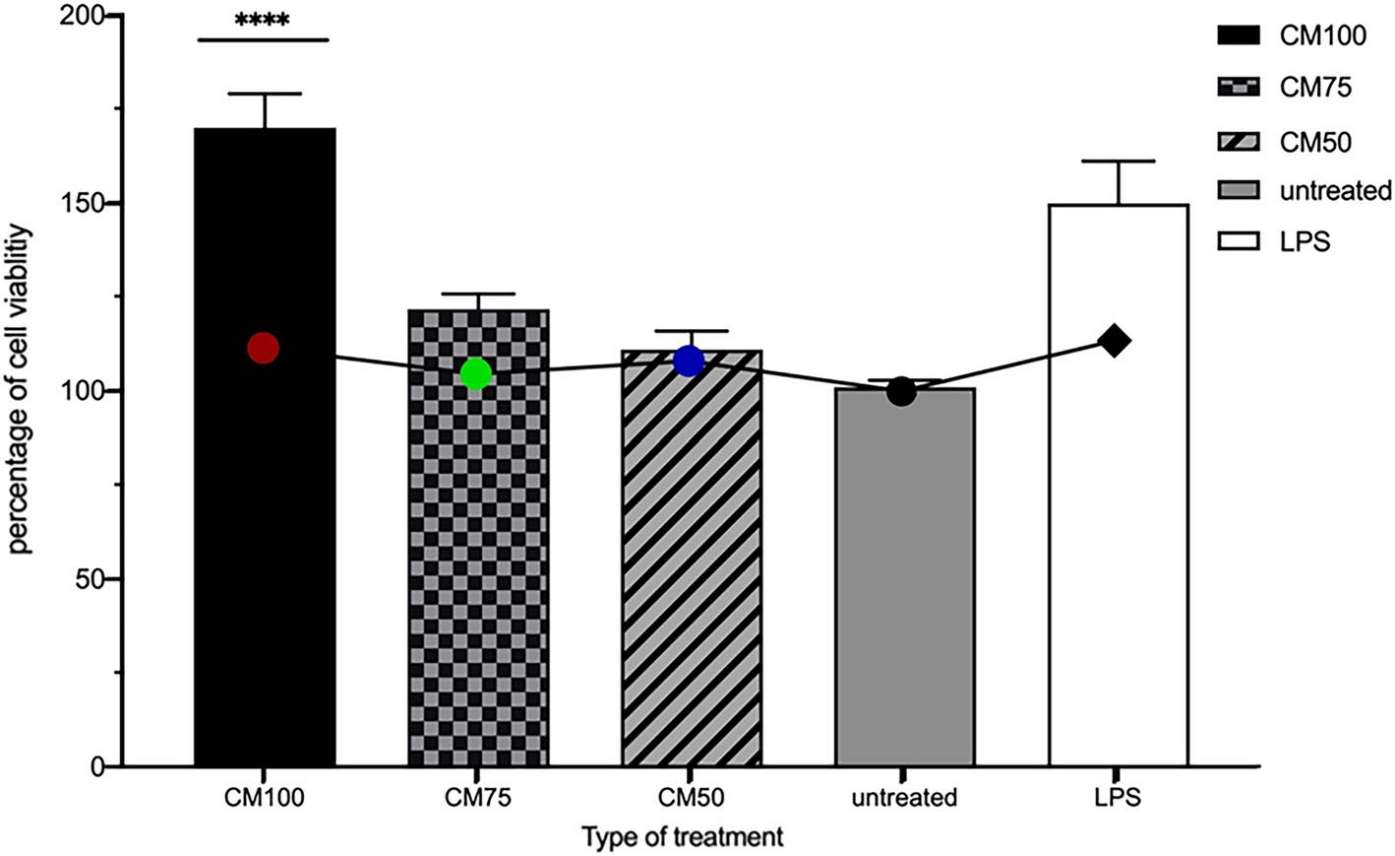
The percentage of viable cells using a cell viability assay after 24 h of CM treatment is represented as a bar graph with different concentrations of CM (50%, 75%, 100%) or with LPS at 1µg/mL as a positive control versus untreated macrophages. This is overlaid with a line graph showing the number of macrophages in treated samples, represented as a percentage compared to untreated macrophages after 24 h of CM treatment. Statistics were performed using a one-way ANOVA test, comparing the groups with the control. Data are presented as means with standard deviations. Level of significance is denoted as follows: *p < 0.05, **p < 0.001, ***p < 0.001, and ****p < 0.0001.

The cytotoxicity of CM at different dilutions (50%, 75%, 100%) was tested using the MTT assay at 3h, 6h, and 24h post-treatment and CM incubation with RAW264.7 murine macrophages. The concentrations of CM (50%, 75%, 100%) had no significant effect on macrophage viability, even when used undiluted (100%), suggesting that the components released by the probiotics or LPS are not toxic to the RAW264.7 murine macrophages (Fig. 1). Therefore, under the experimental conditions used throughout this study, macrophage viability did not appear to be adversely affected by the different concentrations of treatment or controls used. Meanwhile, the 100% CM increased macrophage viability after 3, 6, and most significantly, 24 h of treatment and incubation, as shown in Fig. 1. It should be kept in mind that our sequencing results for the 16S rRNA gene supported the presence of the following bacterial classes: *Bacilli* and *Actinomycetia*. Specifically, it confirmed the content of the *Bifidobacteriaceae* and *Lactobacillaceae* families, as stated on the commercial label. Regarding the cell count results, there were no significant differences in the macrophage cell count 24 h post-treatment with CM (50%, 75%, 100%) and LPS compared to untreated cells (Fig. 2; line graph).

### TNF- α assay

**Figure 3:**
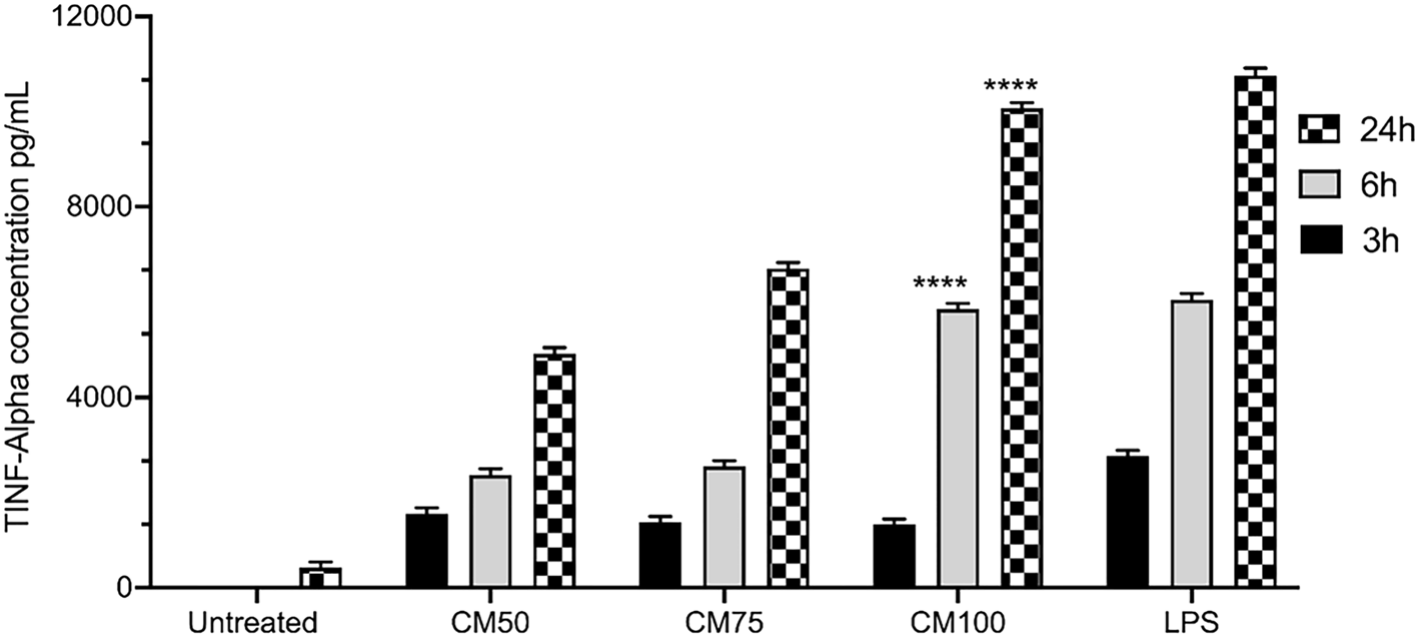
TNF-alpha concentration in cell supernatant from macrophages pretreated overnight with different concentrations of Probiotic Conditioned Media (100%, 75%, 50%) compared with LPS-treated cells and untreated cells at different timeframes (3, 6, and 24 h). Statistics were performed using a one-way ANOVA test, comparing the groups with the control. Data are presented as means with standard deviations. Levels of significance are denoted as follows: *p < 0.05, **p < 0.001, ***p < 0.001, and ****p < 0.0001.

It was observed that the TNF- α content of the supernatant originating from 100% CM exhibited a similar outcome to LPS 1µg/mL, as they stimulated cells and showed a significant release of stimulatory cytokines (Fig. 3).

### Migration assay results

**Figure 4:**
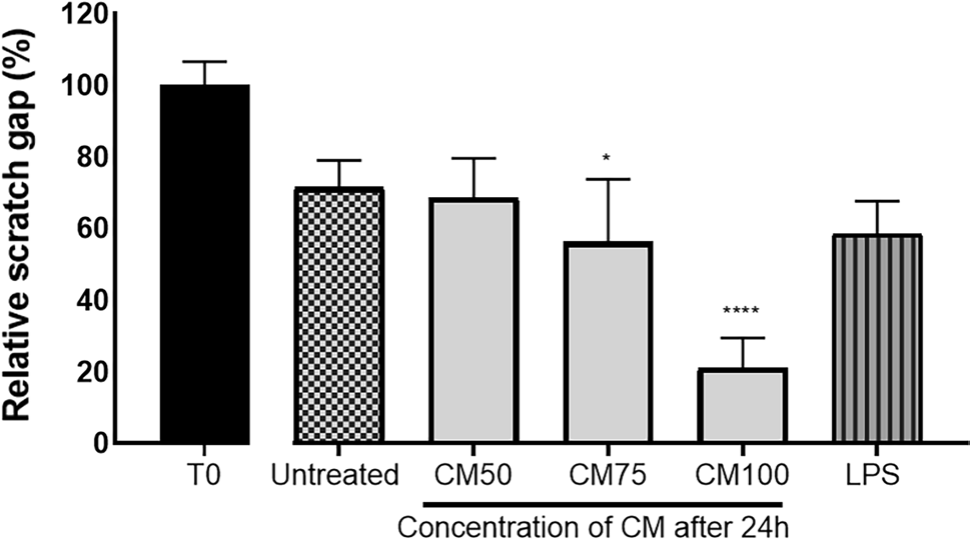
The relative scratch gap percentages of different concentrations of Probiotic Conditioned Media used (CM100, 75, and 50) compared to the control LPS and inactive RAW264.7 over 24, 48, and 72 h. Statistics were performed using a one-way ANOVA test, comparing the groups with the control. Data are presented as means with standard deviations. Levels of significance are denoted as follows: *p < 0.05, **p < 0.001, ***p < 0.001, and ****p < 0.0001.

As shown in Fig. 4, the gap closure of the scratch was significantly enhanced by treating cells with probiotic CM. In addition, the effect of the CM was concentration-dependent compared to negative and positive controls. Hence, gap closure was significantly (*p*-value < 0.0001) better in the CM-100% treated cells compared to the untreated and positive controls.

### Effects of CM-treated RAW264.7 murine macrophage on NO Production

**Figure 5:**
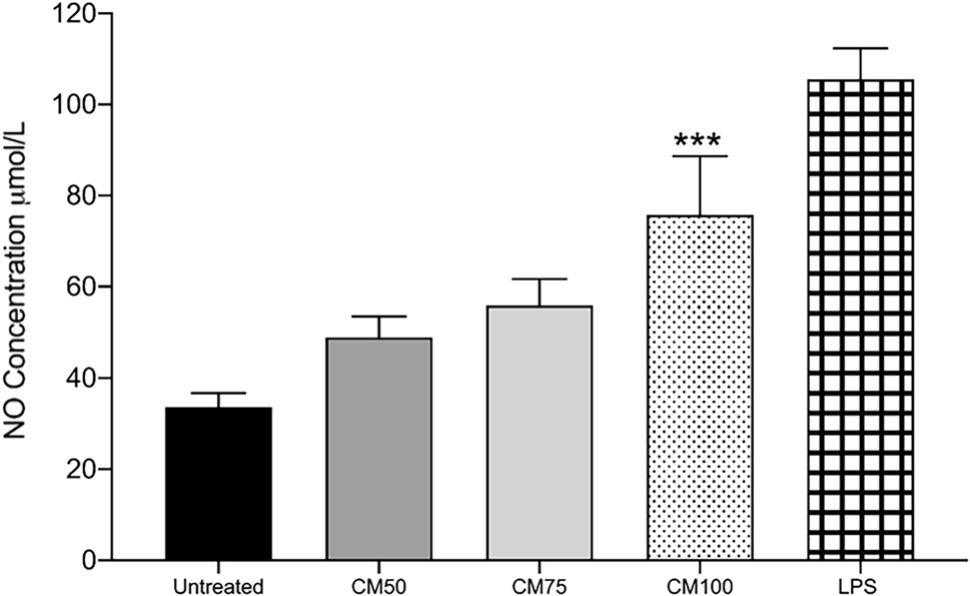
This figure displays the nitric oxide (NO) concentration of supernatant content collected post 24-h treatment of RAW264.7 murine macrophages with different concentrations of Probiotic Conditioned Media (100%, 75%, 50%) compared to LPS 1µg/mL and untreated macrophages as controls. Statistics were performed using a one-way ANOVA test, comparing the groups with the control. Data are presented as means with standard deviations. Levels of significance are denoted as follows: *p < 0.05, **p < 0.001, ***p < 0.001, and ****p < 0.0001.

Treating murine macrophage with CM-100% induced a significant release of NO species (i.e., nitrite content) in supernatant compared to untreated and LPS-treated macrophages (Fig. 5).

### Phagocytosis imaging -fluorescence plate reader results

**Figure 6:**
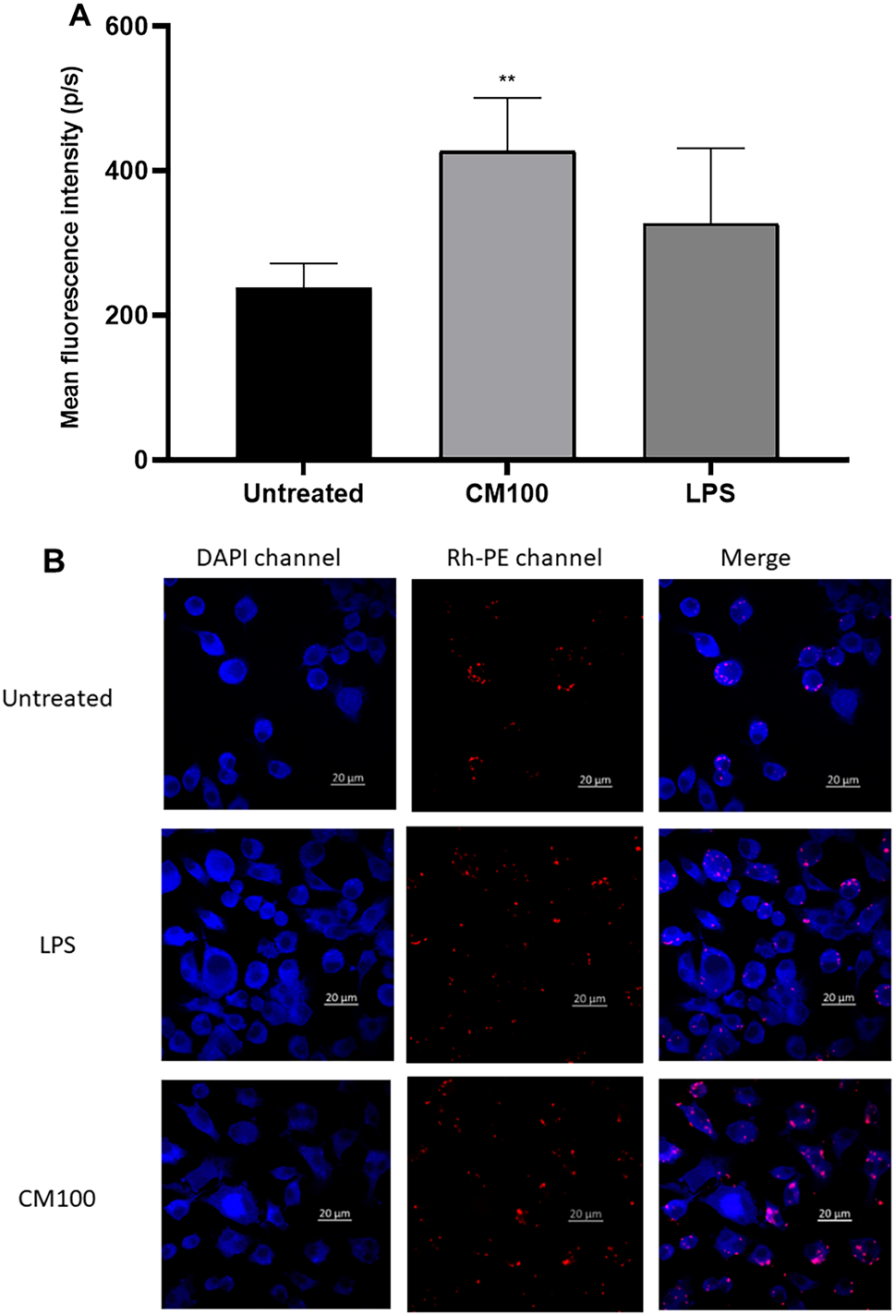
Panel A represents the mean fluorescence intensity of pHrodo™ Red E. coli BioParticles following phagocytosis. Statistics were performed using a one-way ANOVA test, comparing the groups with the control. Data are presented as means with standard deviations. Levels of significance are denoted as follows: *p < 0.05, **p < 0.001, ***p < 0.001, and ****p < 0.0001. Panel B contains confocal microscopy images taken using a confocal LSM 780 at 10 × magnification.

The phagocytosis activity of RAW264.7 cells was substantially enhanced when treating cells with Probiotics CM (Fig. 6). The engulfment process increased nearly 1.8-fold compared to untreated cells. Furthermore, the phagocytic activity was comparable to the positive control (Fig. 6A). Confocal imaging also demonstrated the phagocytosis of the pHrodo™ Red *E. coli* BioParticles.

## Discussion

Probiotics are microorganisms that are widely recognized for their extensive health benefits. Our results suggest that the conditioned medium (CM) supports cell viability (> 80%) and demonstrates an immunostimulatory effect in a concentration-dependent pattern (migration scratch, TNF- α, and phagocytosis assays). The undiluted CM (100%) illustrated a significant (*p*-value < 0.05) stimulatory effect compared to the positive and negative controls. Research has suggested that specific probiotic strains or strain mixtures can significantly impact immune mediators, as demonstrated by their ability to improve conditions such as allergic asthma, atopic dermatitis, and rheumatoid arthritis^34^. Probiotics can modulate both innate and adaptive immune responses through various mechanisms. They can modulate pro-inflammatory cytokine levels, such as TNF-α, IL-6, and IL-1β, and affect the production of NO, iNOS, COX-2, and other immune system parameters^35,36^. Additionally, cell-free conditioned media drawn from cultures of certain probiotics have shown diverse bioactivity, including anti-inflammatory, anticancer, and antiviral effects^37–40^. The activity of the probiotic conditioned medium (CM) may be explained by the excretory compounds produced by the bacteria, which involve probiotic-derived soluble proteins^12,41^, cell membrane polysaccharides, and glycoproteins produced by the probiotic bacteria^42,43^.

The innate immune system plays a crucial role in protecting the human body against foreign substances and pathogens. The main immune cells that are associated with innate immune response are dendritic cells, macrophages, and natural killer (NK) cells^35,44^. Since macrophages are one of the cornerstones of innate immune system activation, we tested the immunomodulatory effect of CM on RAW264.7, demonstrating their impact on macrophage viability and their potential to activate the immune system. Regarding effectiveness, activated macrophages are identified by increased expression of specific mediators such as IL-6, TNF-α, and NO^45,46^.

Previous studies have shown that Bifidobacterial DNA stimulated macrophage expression of NO, IL-1β, IL-6, IL-12p40, and TNF-α^3,47^. Moreover, Nanjundaiah et al. demonstrated the effect of a *Lactobacillus*-conditioned medium on the murine macrophage cell line^28^. Their study suggested that the bacterial-conditioned medium had no effect on macrophage viability, induced nicotinamide adenine dinucleotide phosphate (NADPH) expression, and increased NO production^28^. Our experimental results showed that CM100 was significantly able to upregulate NO production level, which is consistent with the previous study's findings. It is worth mentioning that NO is an M1 macrophage pro-inflammatory marker produced by metabolizing arginine through nitric oxide synthase^48^. Importantly, nitric oxide has been found to target the Th1/Th2 balance of the immune response, resulting in the upregulation of IL-4 (Th2 cytokine) level and a reduction in IL-2 and IFN-γ (Th2 cytokines) expression^49^.

Additionally, it was found that *Lactobacillus* and *Bifidobacterium* increase the production of proinflammatory cytokines, including IL-12 and TNF-α^50,51^. Proinflammatory cytokines, particularly TNF-α, are produced by activated macrophages and act as a promoter of the nuclear factor kappa B (NF-kB) signaling pathway^52^. In turn, NF-kB stimulates many inflammatory genes associated with the activation of M1 macrophages including IL-1β, IL-6, IL-12p40, and cyclooxygenase-2^53^. Rocha-Ramírez et al*.* reported the Lactobacilli effect on simulating proinflammatory bioactive markers such as IL-8, TNF-α, IL-12P70, and IL-1β as well as enhanced phagocytosis activity of macrophages^54^. Moreover, lactic acid-producing bacteria are known to secrete exopolysaccharides that have been found to obtain immunomodulatory potential^55^. Using J774A.1 macrophage cells, exopolysaccharides (from *Bifidobacterium*) were found to induce the secretion of TNF-α at a concentration of 5 µg/mL^56^. In our study, the TNF-α content of the supernatant originating from CM100 showed a comparable outcome to LPS-stimulated cells, thereby displaying a significant release of stimulatory cytokines, such as TNF-α. This may confirm the potential of probiotics to activate the innate immune system.

Mohammed Saeed et al^57–59^. demonstrated that re-epithelialization of wounded skin can be stimulated with L*. rhamnosus GG* lysate and *L. reuteri*. The enhancement in re-epithelialization was likely due to an increase in both keratinocyte migration and proliferation observed with both probiotic lysates. However, the two lysates were not equally effective in promoting cell migration. *L. rhamnosus GG* was much more effective at stimulating migration than *L. reuteri*^57,58^. Other researchers investigated the mechanisms that could improve migration and proliferation, suggesting possible increased expression of transforming growth factor (TGF), vascular endothelial growth factor (VEGF), and fibroblast growth factor (FGF-7)^60^. Gene profiles were examined to define the role of the *L. rhamnosus GG* lysate to upregulate the expression and secretion of Chemokine (C-X-C motif) ligand 2 (CXCL2) from keratinocytes^58^. CXCL2, which has several functions in the wound-healing process of human keratinocyte migration, proliferation, and adhesion, has been reported on in numerous studies^61,62^. It has been shown that CXCL2 could stimulate the secretion of IL-8, which functions as a chemo-attractant for keratinocytes by increasing the expression of the CXCR2 receptor in vitro^63^. These findings align with our study, in which we observed that all concentrations of CM used had faster gap closure than the untreated macrophage control and LPS control (1µg/mL) in the migration assay analysis/wound-healing effect.

Furthermore, the exudates produced by the probiotics used in the study improved the immunostimulatory factors of the RAW264.7 murine macrophages. Particularly, this was observed in cell viability using the MTT assay, production of proinflammatory cytokine tumor necrosis factor-alpha (TNF-α) in macrophages, migration, and phagocytosis assays. For instance, studies have revealed that the cell-free supernatant of probiotics of *Lactobacillus rhamnosus, Lactobacillus fermentum, Pediococcus acidilactici* and *Lactobacillus delbrueckii* subsp, is rich in bioactive compounds such as phenolic and flavonoid^64,65^. These compounds are known for their anti-inflammatory and antioxidant properties, which can influence immune responses^64–66^. Understanding these interactions is crucial in unraveling the potential immunomodulatory mechanisms of probiotics and can have implications for the development of functional foods and supplements aimed at supporting immune health^64–66^.

Though our study sheds light on the immunomodulatory capabilities of multi-species probiotics, it is vital to note some limitations. Specifically, our research did not include a detailed chemical analysis of probiotic secretions, which could provide deeper understanding of the mechanisms directly responsible for the observed immunological parameters. This limitation points to a need for additional research to clarify the specific pathways and signaling mechanisms at play in the interactions between compounds derived from probiotics and immune cells.

## Conclusion

Our study highlights the significant role of multi-species probiotics in enhancing immune function. The immunostimulatory effects observed in this study are likely derived from the secretions and byproducts of various bacterial species that make up the probiotics. However, without a comprehensive chemical analysis of probiotic secretions, our understanding of the direct mechanisms driving these immunomodulatory effects remains limited. Future research should focus on conducting thorough analytical studies to unravel the composition and bioactive properties of probiotic secretions, such as phenolic and flavonoid compounds. This could offer more profound insights into their therapeutic potential. Grasping the link between probiotic secretions and immune functionality can greatly contribute to the development of functional foods and supplements geared towards enhancing overall health and well-being.

## Materials and methods

### Preparation of probiotic conditioned media (CM)

**Figure 7:**
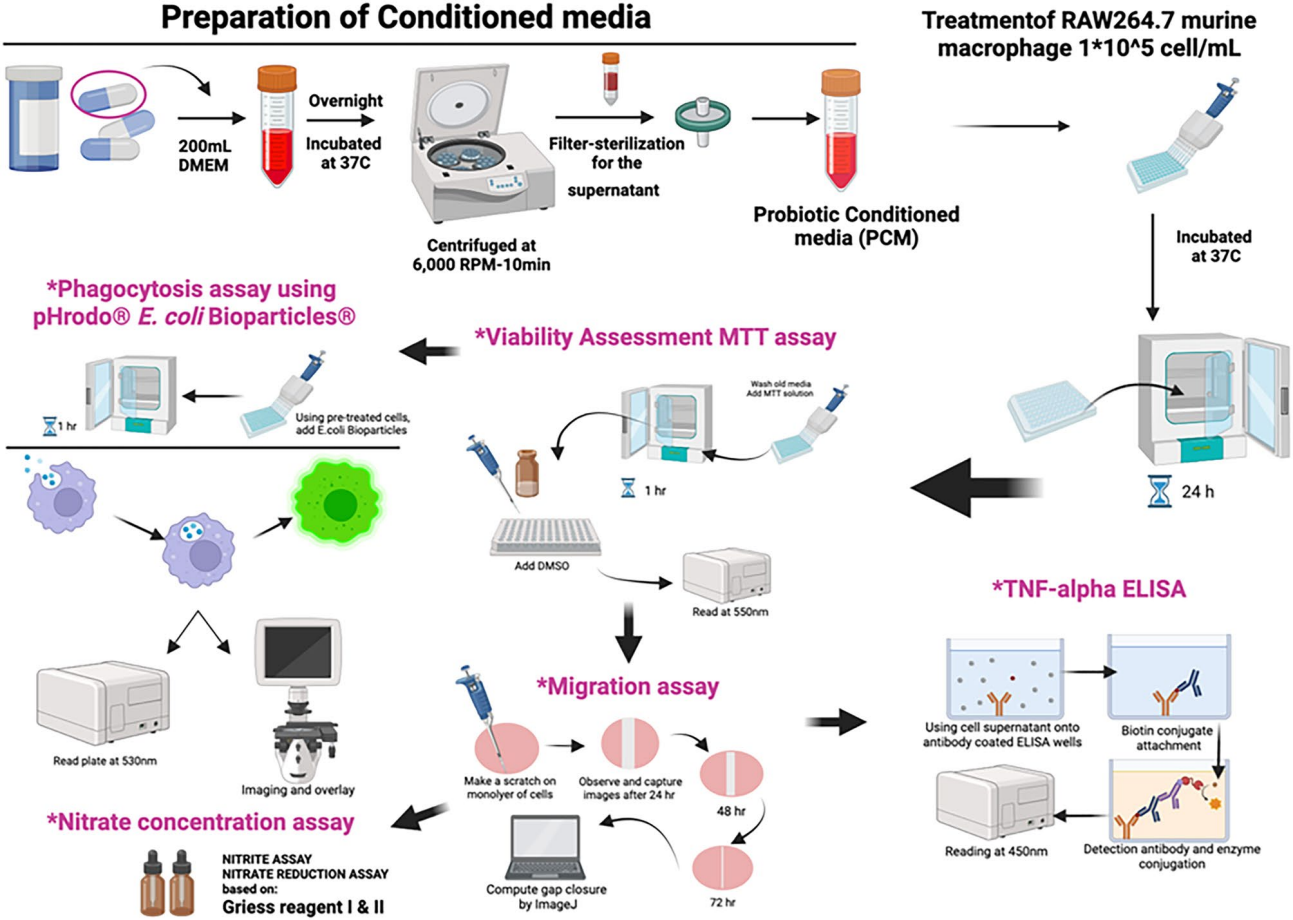
Illustration of the methods used in this work, using Biorender.

Probiotic Conditioned Media (CM) was prepared by dissolving 1 pill content (0.7550g) of the commercially available Advanced Multi-Billion Dophilus™ probiotics (*Lactobacillus acidophilus* 1.25 billion CFU, *Bifidobacterium lactis* 1.25 billion CFU, *Lactobacillus paracasei* 1.25 billion CFU, *Lactobacillus rhamnosus* 1.25 billion CFU) in 200mL of Dulbecco's Modified Eagle Medium (DMEM; Gibco, USA), supplemented with 10% fetal bovine serum (FBS; Gibco, USA). The mixture was kept under aseptic conditions in a sterile sealed glass container for 24 h in 37 °C $\pm 2$ incubator. These conditions allowed a gradient of oxygen concentration, particularly because there was no mixing, which would enable the probiotic to optimally grow under microaerophilic conditions. This was then followed by vigorous mixing for 10 min and overnight incubation at 37 °C. The growth of probiotic bacteria in DMEM has been well established in the literature for most Lactobacillus species and Bifidobacteria^67,68^. In addition, the bacterial growth was further confirmed by noting the turbidity and measuring the optical density (OD600nm = 0.94 ± 0.162). The mixture was then centrifuged at 6000rpm for 15 min and the supernatant was filter-sterilized twice using 0.22µm pore size micro-filters (ExtraGENE). The resulting medium, which was cell-free, is referred to as Probiotic Conditioned Media (CM). The initially obtained cell-free media are considered to be 100% concentration (undiluted), and lower dilutions of 75% and 50% were prepared by using 100% cell-free media diluted with DMEM, supplemented with 10% FBS according to the mentioned ratios. The CM was stored at − 80 °C in a freezer and used as needed (see Fig. 7). It's worth noting that DMEM uses a sodium bicarbonate buffer system (3.7 g/L) to maintain physiological PH. However, the pH was monitored during the experiment using an electrochemical sensor (Hamilton®) connected to a pH meter (HI 2210, HANNA® Instrument), showing a minor pH change of 6.7 ± 0.2.

### Cell culturing and treatment with CM

The murine macrophage cell line RAW264.7 was sourced from the American Type Culture Collection (TIB-71™-ATCC, USA). The macrophages were seeded in DMEM (supplemented with 10% FBS and 1% penicillin/streptomycin (P/S, Gibco™, USA)) and incubated at 37 °C in a humidified 5% CO_2_ incubator. The cells were sub-cultured at 70–80% confluency using 75mL filtered cap flasks. Prior to treatment with CM, RAW264.7 cells were seeded at a density of 1 × 105 cells/mL in 96-well cell culture plates and incubated at 37 °C and 5% CO_2_ in a humidified incubator for 24 h. The cells were then washed with phosphate buffer saline (PBS) and the medium was replaced with 100 µl of CM (50%, 75%, 100%) and re-incubated overnight at 37 °C and 5% CO_2_. *Lipopolysaccharides* from *Pseudomonas aeruginosa* (LPS, Sigma Aldrich, L9143) were used as a positive control (1 µg/mL) and untreated macrophages served as a negative control.

### Viability assessment MTT assay

Following treatment of the RAW264.7 macrophage with CM, cell viability was examined using the 3-(4,5-dimethyl-2-thiazyl)-2,5-diphenyl-2H-tetrazolium bromide (MTT) reduction assay. Briefly, CM-treated RAW264.7 macrophages were washed with PBS three times, and then the MTT solution (5 mg/mL; Sigma Aldrich) was added to each well according to the manufacturer's instructions. The plate was incubated for 1 h under light-protected conditions at 37 °C and 5% CO2. The formazan crystals that formed were dissolved using dimethyl sulfoxide (DMSO, Sigma Aldrich), and the absorbance was measured at 570 nm using a Bio-Tek® microplate reader (Bio-Tek, Germany). Percentage viability was calculated based on the absorbance of media-blanked treatments relative to cells maintained in normal DMEM containing 10% FBS and 1% Penicillin and Streptomycin.

### Cell counting

RAW264.7 macrophages (at a density of 1 × 10^5^ cells/mL) were seeded in DMEM supplemented with 10% FBS and incubated in 6-well plates for 24 h prior to the assay. The next day, the media was removed, cells were washed three times with PBS, and then treated accordingly with CM (50%, 75%, 100%) at a volume of 1ml per well. LPS was used as a positive control (1µg/mL), and untreated macrophages served as a negative control. After 24 h of treatment, the treatment media was removed; each well was washed three times again with PBS, the cells were scraped using a cell scraper with 1ml of DMEM supplemented with 10% FBS, and then counted using a hemocytometer.

### Cell migration scratch assay

RAW264.7 macrophages (at a density of 1 × 10^5^ cells/mL) were seeded in 96-well plates 24 h prior to the assay. A scratch was made in each well using a 10 µl micro-pipette, and then each well was washed with 100 µl of phosphate buffer saline (PBS, Gibco, USA). CM (50%, 75%, 100%) was added at 100 µl per well to the scratched areas. LPS was used as a positive control (at 1 µg/mL), and untreated macrophages were used as a negative control. The plate was then incubated at 37 °C in a humidified 5% CO_2_ incubator, and the closure of the gaps was monitored using an inverted lens microscope at 24, 48, and 72-h intervals. The ImageJ program was used to calculate gap closure.

### Cytokine release assessment

RAW264.7 cells (at a density of 1 × 10^5^ cells/mL) were treated with different concentrations of CM (50%, 75%, 100%), alongside positive and negative controls. Supernatants were collected after 6 h of incubation and the level of TNF-α in the cell-free supernatant was assessed using ELISA according to the manufacturer's instructions. The production of TNF-α was demonstrated following the steps of applying the coating antibody, treating each sample or standard, adding the biotin conjugate and detection antibody, applying the substrate conjugate, performing the enzyme conjugation, initiating colorization, and finally reading the results at 450 nm.

### Phagocytosis assay and imaging

The phagocytosis assessment was conducted using pHrodo™ Red *E. coli* BioParticles™ (Abcam, USA). After treating RAW 267.4 cells with 100% CM for 24 h (this concentration was selected based on the results of the initial screening test), the cells were washed twice with PBS, and then 5 µl of red fluorescence *E. coli* was added to each well and incubated at 37 °C, 5% CO_2_ for 1 h. These *E. coli* BioParticles™ are tagged with pHrodo dye, which significantly fluoresces as the pH decreases from neutral to acidic following engulfment. The wells were washed twice again with PBS and the fluorescence was measured using a Bio-Tek® microplate reader at.

For fluorescence imaging, RAW 246.7 cells were seeded overnight on coverslips found in a 6-well plate at 1 × 10^5^ of viable cells/well. The following day, the cells were treated with CM and *E. coli* BioParticles™. For staining, two drops of Mounting Medium with DAPI solution (AB104139, Abcam®) were added to the slide and covered with a coverslip. The imaging process was carried out using a confocal LSM 780 (ZEISS, Germany), with the DAPI channel set to 410–585 and the Rh-PE to 566–691.

### Nitrite concentration assay

Nitrite quantification was carried out according to the manufacturer's protocol (Parameter™, R&D systems, USA). In this assay, nitric oxide concentrations were determined based on the enzymatic conversion of nitrate to nitrite by nitrate reductase. This reaction is followed by the colorimetric detection of nitrite as an azo-dye product of the Griess Reaction. The process involved diluting 50 µl of cell supernatant five times and subjecting it to two consecutive assays. The nitrite assay was performed first by adding 50 µl each of Griess I and Griess II, then incubating it at room temperature for 10 min. The absorbance was then measured at 550 nm on a plate reader. The nitrate reductase assay was performed by adding 25 µl of NADH and 25 µl of nitrate reductase, then incubating it at 37 °C for 30 min. Next, 50 µl each of Griess I and Griess II were added, and the sample was incubated at room temperature for 10 min. The absorbance was then measured at 550nm on a plate reader. The calculation of nitric oxide content was based on standard graph equations and subtracting the concentration of the nitrite assay from the nitrate reductase concentration.

### Bacterial DNA extraction and DNA sequencing

Bacterial DNA extraction was performed according to the QIAamp DNA extraction miniKit protocol (Germany). The 16S rRNA gene V4 variable region was amplified using PCR primers 27F-519R in a 30-cycle PCR using the HotStarTaq Plus Master Mix Kit (Qiagen, USA) with the following conditions: 95 °C for 5 min, 30 cycles of 95 °C for 30 s, 53 °C for 40 s, and 72 °C for 1 min, then a final elongation step at 72 °C for 10 min. Samples were multiplexed using unique dual indices and pooled together in equal proportions based on their molecular weight and DNA concentrations. Pooled samples were purified using calibrated Ampure XP beads. The pooled and purified PCR products were then used to prepare an Illumina DNA library. Sequencing was performed at MR DNA (Shallowater, TX, USA) on a MiSeq, following the manufacturer's guidelines. Sequence data were processed and analyzed using the MR DNA analysis pipeline (MR DNA, Shallowater, TX, USA). Final zOTUs were taxonomically classified using BLASTn against a curated database derived from NCBI (National Center for Biotechnology Information, USA).

### Statistical analysis

The results were statistically analyzed using GraphPad Prism 6 (GraphPad Software, Inc., San Diego, CA, United States). A one-way analysis of variance (ANOVA) was used to compare the means of three replicates across two or more independent groups, and Tukey's multiple comparison test was used as a post hoc test. Differences were considered significant when *p*-values were less than 0.05. Data were presented as means and standard deviations. The significance level was denoted as follows: *p < 0.01, **p-value < 0.001, ***p-value < 0.001, and ****p-value < 0.0001.

## Data Availability

The datasets generated and/or analyzed during the current study are available from the corresponding author upon reasonable request.
